# The pluripotency factor NANOG promotes the formation of squamous cell carcinomas

**DOI:** 10.1038/srep10205

**Published:** 2015-05-19

**Authors:** Adelaida R. Palla, Daniela Piazzolla, Noelia Alcazar, Marta Cañamero, Osvaldo Graña, Gonzalo Gómez-López, Orlando Dominguez, Marta Dueñas, Jesús M. Paramio, Manuel Serrano

**Affiliations:** 1 Tumour Suppression Group, Spanish National Cancer Research Centre (CNIO), Madrid 28029, Spain; 2 Histopathology Core Unit, Spanish National Cancer Research Centre (CNIO), Madrid 28029, Spain; 3 Bioinformatics Unit, Spanish National Cancer Research Centre (CNIO), Madrid 28029, Spain; 4 Genomics Core Unit, Spanish National Cancer Research Centre (CNIO), Madrid 28029, Spain; 5 Molecular Oncology Unit, CIEMAT, Madrid 28040, Spain; 6 Oncogenomic Unit, Institute of Biomedical Research, University Hospital “12 de Octubre”, Madrid 28040, Spain

## Abstract

NANOG is a key pluripotency factor in embryonic stem cells that is frequently expressed in squamous cell carcinomas (SCCs). However, a direct link between NANOG and SCCs remains to be established. Here, we show that inducible overexpression of NANOG in mouse skin epithelia favours the malignant conversion of skin papillomas induced by chemical carcinogenesis, leading to increased SCC formation. Gene expression analyses in pre-malignant skin indicate that NANOG induces genes associated to epithelial-mesenchymal transition (EMT). Some of these genes are directly activated by NANOG, including EMT-associated genes *Zeb1, Zeb2*, *Twist1, Prrx1* and *miR-21*. Finally, endogenous NANOG binds to the promoters of theses genes in human SCC cells and, moreover, NANOG induces EMT features in primary keratinocytes. These results provide *in vivo* evidence for the oncogenic role of NANOG in squamous cell carcinomas.

NANOG is a homeodomain containing transcription factor that together with OCT4 and SOX2 constitute the core regulators of pluripotency in mouse embryonic stem cells^1^,^2^,^3^. Cancer cells present some similarities with embryonic stem cells, including unlimited proliferation and self-renewal, and the expression of pluripotency genes, such as NANOG, OCT4 or SOX2^4^. In the particular case of SOX2, it is well established that it is a driver of oncogenesis in several cancer types^5^,^6^,^7^,^8^.

The oncogenic potential of NANOG has been demonstrated using multiple types of assays, including promotion of proliferation, xenograft growth, migration and invasion, chemoresistance, and cancer stem cell properties^9^,^10^. Also, transgenic overexpression of *Nanog* in MMTV-Wnt-1 mice accelerates mammary tumorigenesis and metastasis^11^. Distinctively, NANOG is frequently overexpressed in human squamous cell carcinomas (SCCs) and its expression correlates with malignancy and chemoresistance^12^,^13^,^14^,^15^,^16^. However, there is a lack of *in vivo* studies addressing the impact of NANOG on the formation of SCCs and the mechanisms involved. In this study, (1) we use an epithelial inducible transgenic mouse model to analyze the effect of NANOG in the generation of SCCs, and (2) we identify NANOG as a cell autonomous activator of EMT in epithelial cells.

## Results

### NANOG promotes proliferation in the epidermis

**Figure 1:**
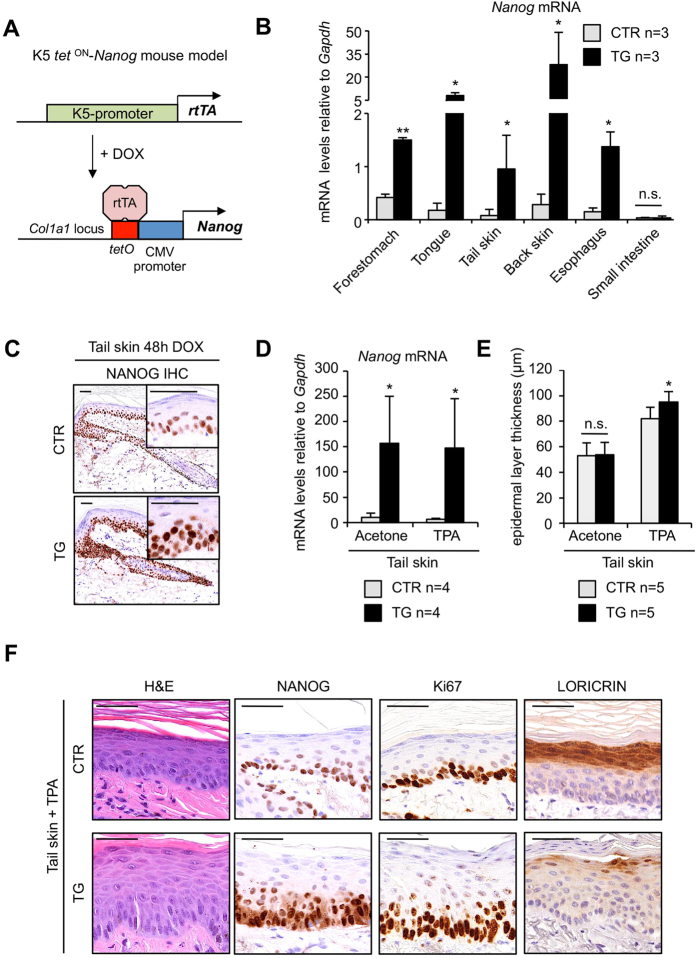
*Nanog*-inducible mouse model. (**A**) Schematic representation of the *K5-rtTA;Col1a1::tetO-Nanog* mouse model. The reverse transactivator (rtTA) is under the control of the bovine cytokeratin 5 (K5) promoter. A cassette containing the *Nanog*-cDNA is under the control of the doxycycline (DOX)-responsive promoter (*tetO*), which was inserted downstream of the *Col1a1* locus. Arrows indicate transcriptional start sites. (**B**) Relative *Nanog* mRNA levels in the indicated organs determined by qRT-PCR. *Nanog*-inducible mice (n = 3) containing both transgenes (*K5-rtTA*^*tg/.*^;*Col1a1*^*tetO-Nanog*/+^; abbreviated as TG) and control mice (n = 3) lacking the transactivator (*Col1a1*^*tetO-Nanog*/+^; abbreviated as CTR) were analyzed 48 hr after administration of doxycycline (DOX) (2 mg/ml) in the drinking water. (**C**) Immunohistochemistry (IHC) for NANOG of paraffin-embedded sections of tail skin from CTR and TG mice treated as indicated in (**B**). Two magnifications are shown for each tissue (bars correspond to 50 μm). (**D**) *Nanog* mRNA levels normalized to *Gapdh* in CTR and TG tail skin topically treated 4 times, at 48 hr intervals, with 12-*O*-tetradecanoylphorbol 13-acetate (TPA) or acetone as a control. Mice (n = 4) were exposed to doxycycline (2 mg/ml) in their drinking water 48 hr prior to TPA treatment and throughout the remaining protocol. (**E**) Epidermal thickness (basal and suprabasal layers) of CTR and TG mice (n = 5) treated as in (**D**). (**F**) Representative hematoxylin and eosin (H&E) staining and immunohistochemistry (IHC) for NANOG, Ki67 and LORICRIN of TPA-treated CTR and TG mice as indicated in (**D**) (bars correspond to 50 μm). Bars in (**B**, **D**, **E**) correspond to mean ± SD of the indicated number of mice (n). Statistical significance was determined by the two-tailed Student’s t test: (*) *p* < 0.05; (**) *p* < 0.01.

We have previously reported that NANOG is expressed in the basal layer of stratified epithelia in normal mice, including the skin^16^. In general, tumorigenesis of stratified epithelia gives rise to squamous cell carcinomas (SCCs) and, remarkably, NANOG is frequently overexpressed in human and mouse SCCs^12^,^13^,^14^,^15^,^16^. To address the *in vivo* role of NANOG in oncogenesis, we have used a tissue specific doxycycline-inducible *Nanog* transgenic mouse model. This model is similar to a previously described *Oct4*-inducible mouse model^17^, in which a cassette containing a doxycycline-responsive element controlling *Nanog* expression is inserted downstream of the collagen 1a1 locus (*Col1a1::tetO-Nanog*)^16^. This transgenic mouse model was crossed with another available mouse model (*K5-rtTA)*, which has the bovine keratin 5 (K5) promoter directing the expression of the reverse tetracycline transactivator gene^18^ (Fig. 1A). The combination of these transgenes (*K5-rtTA;Col1a1::tetO-Nanog*) provides a Tet-ON tool that allows the inducible expression of *Nanog* in the basal layer of the skin, which includes the stem cell compartments^19^.

We first examined whether transgenic *Nanog* was specifically expressed in the basal layer of stratified epithelium. We treated adult *K5-rtTA*^*tg/.*^;*Col1a1*^*tetO-Nanog*/ + ^(abbreviated as TG) and their control littermates lacking the rtTA transactivator *Col1a1*^*tetO-Nanog*/ + ^(abbreviated as CTR) with doxycycline (DOX) in the drinking water (2 mg/ml) for a period of 48 hours. We observed *Nanog* overexpression selectively in tissues containing stratified epithelia, such as, forestomach, tongue, skin, and esophagus. As a negative control, we could not detect overexpression of transgenic *Nanog* in small intestine (non-stratified epithelial tissue) (Fig. 1B).

We also performed immunohistochemistry (IHC) of NANOG to determine its expression in the basal layer of the skin (Fig. 1C). Consistent with our previous study, NANOG was detected by IHC in CTR mice^16^. Also, as intended, after 48 hr treatment with DOX, TG mice showed stronger NANOG staining in the basal layer of the skin compared to the CTR (Fig. 1C). Of note, NANOG overexpression did not produce detectable histological alterations in the tail skin after 48 hr of DOX (Fig. 1C) or after continuous DOX administration during 9 months (Supplementary Figure S1). Therefore, at the levels of NANOG overexpression achieved with the *K5*-*rtTA* system, NANOG alone is not sufficient to alter the homeostasis of the skin.

To investigate the effect of NANOG in the context of mitogenic and inflammatory stimulation, we topically treated the tail skin of CTR and TG mice with 12-*O*-tetradecanoylphorbol 13-acetate (TPA). Four applications of TPA at 48 hr intervals resulted in augmented epidermal thickness compared to CTR mice (Fig. 1D,E). This hyperplasia was accompanied by a clear increase in proliferating keratinocytes (detected by Ki67 staining) in the basal and suprabasal layers of TG mice compared to CTR ones, as well as a decrease of the differentiation marker LORICRIN (Fig. 1F). The mitogenic activity of NANOG in the epidermis is in agreement with our previous observations upon whole-body NANOG induction^16^ and also with the recent finding that NANOG is activated by the TPA-target PKCε^20^. We conclude that NANOG has the capacity to augment epidermal proliferation and to impair differentiation under conditions of mitogenic stimulation.

### NANOG promotes squamous cell carcinoma (SCC) formation

**Figure 2:**
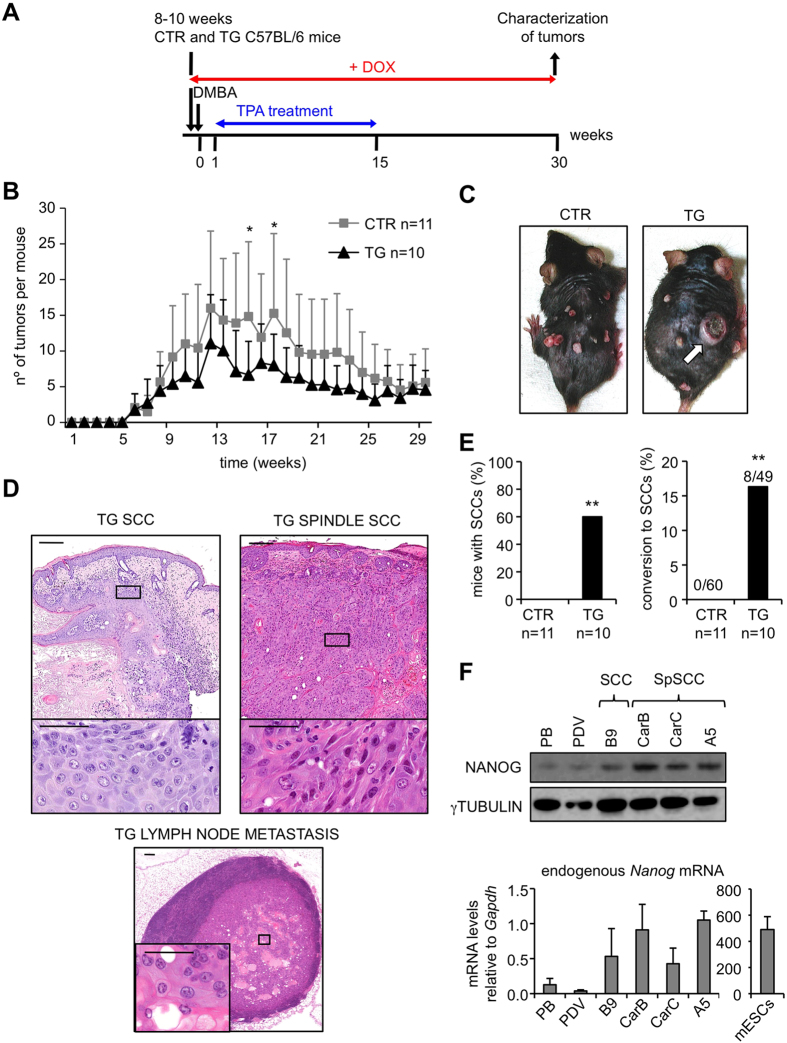
*Nanog* overexpression promotes skin squamous cell carcinoma. (**A**) Experimental layout for two-stage chemically-induced carcinogenesis. CTR and TG mice (see Fig. 1A) were treated with doxycycline (DOX) in their drinking water (2 mg/ml) during the entire protocol. DMBA was applied 48 hr after initiation of DOX treatment. TPA application started the following week, twice a week during 15 weeks. Tumors were counted and measured weekly. Histological analysis was performed at the humane endpoint or at the end of the experiment (30 weeks). (**B**) Average number of tumors per mouse during the treatment. Statistical significance was determined for each time point by two-tailed Student’s t test: (*) p < 0.05. (**C**) Representative images of DMBA/TPA treated mice. Arrow indicates a squamous cell carcinoma. (**D**) Hematoxylin and eosin (H&E) staining of TG carcinomas and lymph node metastasis. Two magnifications are shown for each tissue (bars correspond to 200 μm in the low magnification pictures and to 50 μm in the high magnification pictures). (**E**) Left, percentage of CTR and TG mice with squamous cell carcinomas (SCCs) at the end of the treatment. Right, conversion rate showing the number of SCCs relative to the total number of tumors. Statistical significance was assessed by the Fisher’s exact test: (**) *p* < 0.01. (**F**) Top, immunoblots of NANOG using total lysates from the indicated cell lines. γTUBULIN was used as a loading control. Bottom, endogenous *Nanog* mRNA levels from the indicated cell lines. Mouse ESCs were used as a positive control for *Nanog* expression. mRNA levels were normalized by *Gapdh* levels. Values correspond to the mean ± SD of two independent isolates (n = 2), each one analyzed in triplicate. SpSCC, spindle SCC.

Recent reports have shown that NANOG is overexpressed in human and mouse SCCs^12^,^13^,^14^,^15^,^16^. To address whether high levels of NANOG can contribute to tumorigenesis, we induced skin tumors using the classical two-stage chemical carcinogenesis protocol based on the mutagen 7,12-dimethylbenz(a)anthracene (DMBA) and the promoter TPA^21^. We used CTR and TG mice in a C57BL/6 background, which is known to be resistant to carcinoma formation^22^, and administered DOX throughout the entire protocol (Fig. 2A). There were no significant differences in the kinetics of papilloma formation (Fig. 2B), or in the total number and size of papillomas at the end of the experiment (Supplementary Figure S2A and B). Interestingly, at around 20 weeks of treatment, tumors resembling SCCs started to appear exclusively in the TG mice (Fig. 2C). In many cases (~40%), TG mice had to be sacrificed before the finalization of the observation period because carcinomas were larger than 1.5 cm (humane endpoint) (Supplementary Figure S2C). All tumors were characterized at the end of the protocol or at the humane endpoint. As expected for the C57BL/6 background, all CTR tumors were benign papillomas. In contrast, a remarkable fraction of tumors (>15%) from the TG mice had progressed to SCCs (Fig. 2D,E). We confirmed NANOG overexpression in the TG papillomas and carcinomas (Supplementary Figure S2D and E). We also noticed that the endogenous *Nanog* gene was upregulated in TG papillomas and carcinomas (Supplementary Figure S2D), suggesting that transgenic *Nanog* may upregulate the expression of the endogenous *Nanog* allele. Interestingly, some *Nanog-*SCCs presented areas with spindle histology (Fig. 2D) and in one case we have detected metastasis to a lymph node (Fig. 2D). The protein and mRNA levels of NANOG were similarly high in the metastasis and the SCC from the same animal (Supplementary Figure S2F and G).

To further explore the association between NANOG and skin tumorigenesis, we analyzed NANOG protein and mRNA levels in different cell lines previously obtained from DMBA/TPA-induced tumors. Spindle SCC-derived cell lines (CarB, CarC and MSC11A5) and non-spindle SCC-derived cell line (MSC11B9) expressed higher levels of NANOG, compared to cell lines derived from papillomas (PB) or transformed keratinocytes (PDV) (Fig. 2F).

Together, these results demonstrate that NANOG can promote malignant progression to skin squamous cell carcinoma.

### NANOG induces EMT targets in skin papillomas

**Figure 3:**
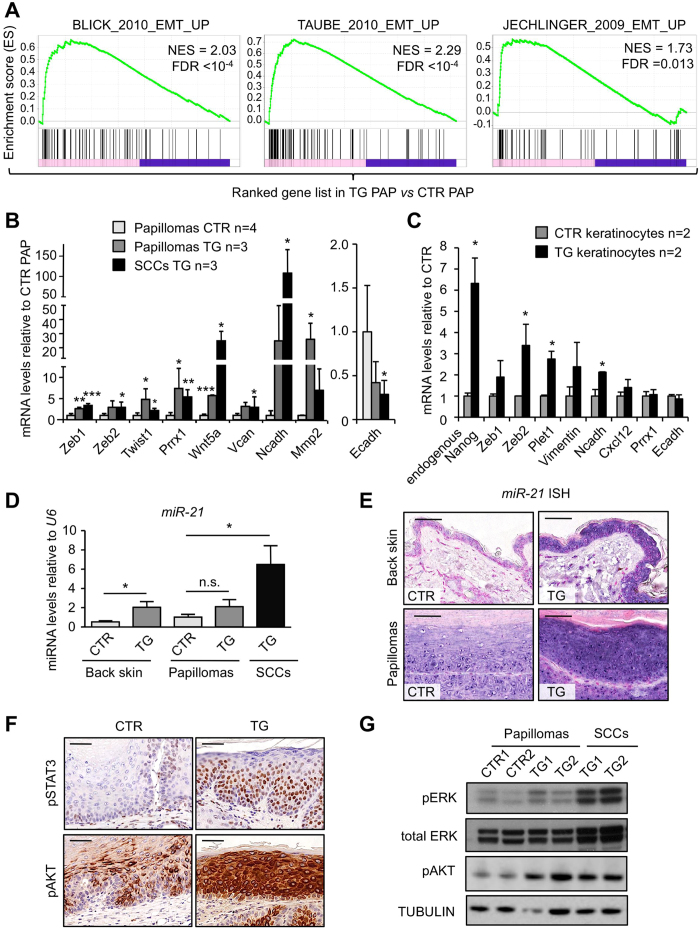
NANOG promotes epithelial-mesenchymal transition (EMT) *in vivo*. (**A**) Gene set enrichment data (GSEA) showing the enrichment of three published EMT gene signatures^23^,^24^,^25^ in TG papillomas as compared with CTR papillomas. NES: normalized enrichment score. The false discovery rate (FDR; q-value) is indicated. (**B**) Relative gene expression (fold change) in CTR and TG papillomas and SCCs of EMT related genes analyzed by qRT-PCR. mRNA levels were normalized by *Gapdh* levels and then expressed as the ratios with respect to CTR papillomas (CTR PAP). (**C**) Relative mRNA levels of the indicated genes in primary keratinocytes extracted from CTR and TG newborn pups (n = 2) and treated during 48 hr with DOX. mRNA levels were normalized by *Gapdh* levels and then expressed as the ratios with respect to CTR keratinocytes (shown as fold change). (**D**) *miR-21* levels normalized to *U6* in CTR (n = 4) and TG (n = 4) back skin; CTR (n = 4) and TG (n = 3) papillomas; and TG (n = 3) SCCs. (**E**) *In situ* hybrdization (ISH) of *miR-21* in representative CTR and TG back skin and papillomas (bars correspond to 50 μm). (**F**) IHC of phospho-STAT3 (Tyr705) and phospho-AKT (Ser473) in representative CTR and TG papillomas (bars correspond to 50 μm). (**G**) Immunoblots of phospho-ERK (Thr202/Tyr204) and phospho-AKT (Ser473) in lysates from back skin, papillomas and SCCs from CTR and TG mice. Total ERK and TUBULIN were used as a loading controls. Bars in (**B**,**C**,**D**) correspond to mean ± SD of the indicated number of samples (n), each from different mouse. Statistical significance was determined by the two-tailed Student’s t test: n.s., non significant; (*) *P* < 0.05; (**) *P* < 0.01; (***) *P* < 0.001. In (**B**), statistic analysis is performed relative to CTR papillomas.

To obtain mechanistic insight on how NANOG promotes skin tumorigenesis, we performed RNA-seq of CTR (n = 4) and TG (n = 3) papillomas. This analysis revealed a small number of differentially-expressed genes upregulated by transgenic *Nanog* expression (97 genes; see **Table S1**). We noted the presence of a high number of genes (up to 13 genes) previously associated to epithelial-mesenchymal transition (EMT) (**Table S1**). This was confirmed by gene set enrichment analysis (GSEA) of a number of published EMT signatures^23^,^24^,^25^ (Fig. 3A). We validated by qRT-PCR the upregulation of EMT drivers, such as *Zeb1*, *Zeb2*, *Twist* and *Prrx1*, and EMT mediators, such as *Wnt5a*, *Vcan*, *Ncadh* and *Mmp2* both in *Nanog-*papillomas and in *Nanog-*SCCs (Fig. 3B). Of note, downregulation of E-cadherin was also observed by qRT-PCR and IHC (Fig. 3B and Supplementary Figure S3A), as well as upregulation of the mesenchymal marker VIMENTIN in *Nanog*-SCCs (Supplementary Figure S3A).

Based on the above findings, we asked whether NANOG had the ability to initiate an EMT program in primary keratinocytes. For this, we extracted keratinocytes from CTR and TG neonates and induced *Nanog* expression by *in vitro* treatment with DOX for 48 hr (Supplementary Figure S3B). Interestingly, NANOG was able to upregulate a subset of EMT mediators in primary keratinocytes as soon as 48 hr after induction (Fig. 3C).

MicroRNAs (miRs) have been also implicated as important regulators of EMT and malignancy, being particularly important miR-21, as inducer of EMT, and the miR-200 and miR-34 families, as negative regulators of EMT^26^,^27^,^28^,^29^. We examined the expression of these miRNAs in normal skin, papillomas and carcinomas. Interestingly, by qRT-PCR and *in situ* hybridization (ISH), we found that miR-21 was significantly upregulated in the back skin, papillomas and SCCs after induction of *Nanog* for 30 weeks (Fig. 3D,E and Supplementary Figure S2G). In contrast, miRNAs from the miR-200 and miR-34 families were essentially unchanged between CTR and TG samples (Supplementary Figure S3C). It has been reported that NANOG requires phosphorylated STAT3 (pSTAT3) for efficient upregulation of *miR-21* in head and neck SCC cell lines^13^. In this regard, we found that NANOG-overexpressing papillomas also express high levels of pSTAT3 (Fig. 3F), thereby lending support to the above-mentioned link between NANOG and pSTAT3 in SCCs. The biological effects of miR-21 go beyond EMT and include the activation of oncogenic signaling pathways, as reflected by an increase in the phosphorylated forms of AKT (pAKT) and ERK (pERK)^26^,^28^,^30^. In agreement with this, we observed increased levels of pAKT and pERK (Fig. 3F,G) in *Nanog*-driven tumors.

Together, these observations indicate that NANOG drives SCC tumorigenesis in association with the upregulation of EMT drivers, notably including *Zeb1*, *Zeb2*, *Twist*, *Prrx1* and *miR-21*.

### NANOG induces cancer stem cell markers in skin papillomas

**Figure 4:**
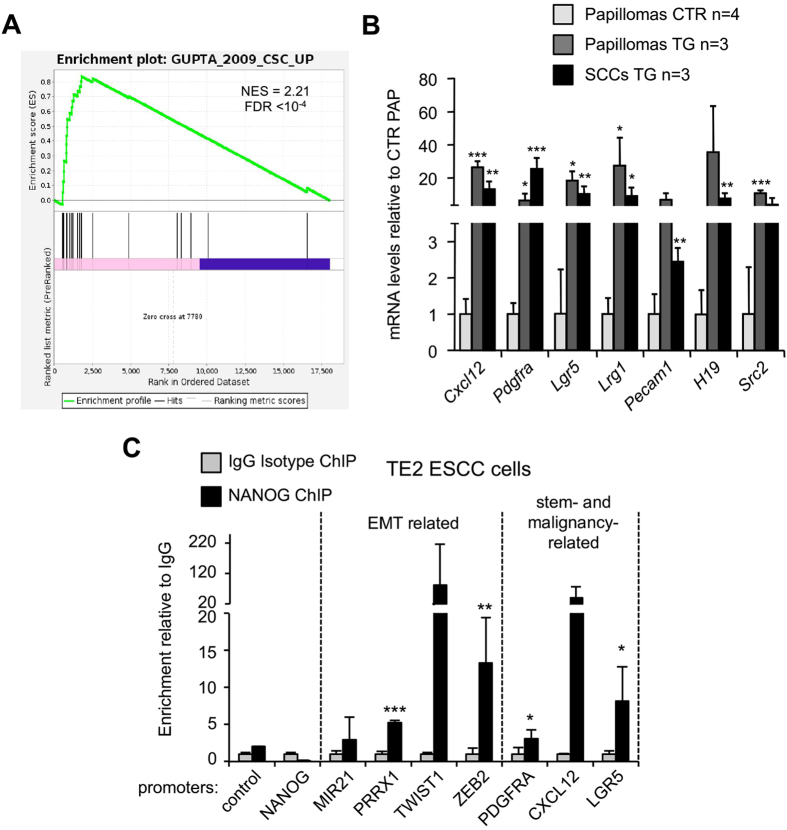
NANOG directly regulates EMT mediators. (**A**) Gene set enrichment data (GSEA) showing the enrichment of a published breast cancer stem cell signature^31^ in TG papillomas as compared with CTR papillomas. NES: normalized enrichment score. The false discovery rate (FDR, q-value) is indicated. (**B**)Relative gene expression in CTR and TG papillomas and TG SCCs of stemness related genes analyzed by qRT-PCR. mRNA levels were normalized by *Gapdh* levels and, then expressed as the ratios with respect to CTR papillomas (shown as fold change). Values correspond to mean ± SD of the indicated number of samples (n), each from a different mouse. Statistical significance was determined by the two-tailed Student’s t test relative to CTR papillomas. (**C**) Chromatin immunoprecipitation (ChIP) of NANOG and goat IgG in TE2 esophageal SCC (ESCC) cells. Values were first normalized to the input values, then and expressed as the ratios with respect to the IgG control (shown as fold change). Values correspond to mean ± SD of two independent biological replicates (n = 2). Control, genomic region that does not bind NANOG. Statistical significance in (**B**,**C**) was determined by the two-tailed Student’s t test. (*) *P* *<* 0.05; (**) *P* *<* 0.01; (***) *P* < 0.001.

Examination of the list of upregulated genes in *Nanog*-papillomas also revealed the presence of genes previously related to cancer stem cell properties (**Table S1**). Gene set enrichment analysis (GSEA) using a cancer stem cell signature from breast cancer^31^ confirmed this idea (Fig. 4A). A number of genes related to cancer stem cells were validated by qRT-PCR, including *Cxcl12/Sdf-1, Pdgfra*, *Lgr5*, *Lrg1*, *Pecam1/CD31*, *H19* and *Src2*, which were all significantly upregulated in *Nanog-*papillomas and *Nanog-*SCCs relative to control papillomas (Fig. 4B).

### NANOG directly binds to the promoters of its target genes in SCC cells

We took advantage of the ENCODE project^32^ to examine ChIP-seq data of NANOG in human ESCs. Remarkably, the above-mentioned *Nanog*-upregulated genes involved in EMT or in cancer stem cells presented NANOG binding sites in human ESCs according to the ENCODE data (see Supplementary Methods), and we confirmed this in human embryonal carcinoma NTERA2 cells (Supplementary Figure S4). We wondered if endogenous NANOG was also bound to these genes in SCC cells. To test this, we performed ChIP in the human esophageal SCC (ESCC) cell line TE2, which is known to have high endogenous levels of NANOG^16^. Interestingly, we observed direct binding of endogenous NANOG to the promoters of EMT mediators *PRRX1* and *ZEB2*, as well as, to stem- and malignancy-related genes *PDGFRA* and *LGR5* (Fig. 4C).

### NANOG promotes EMT in a cell-autonomous manner

**Figure 5:**
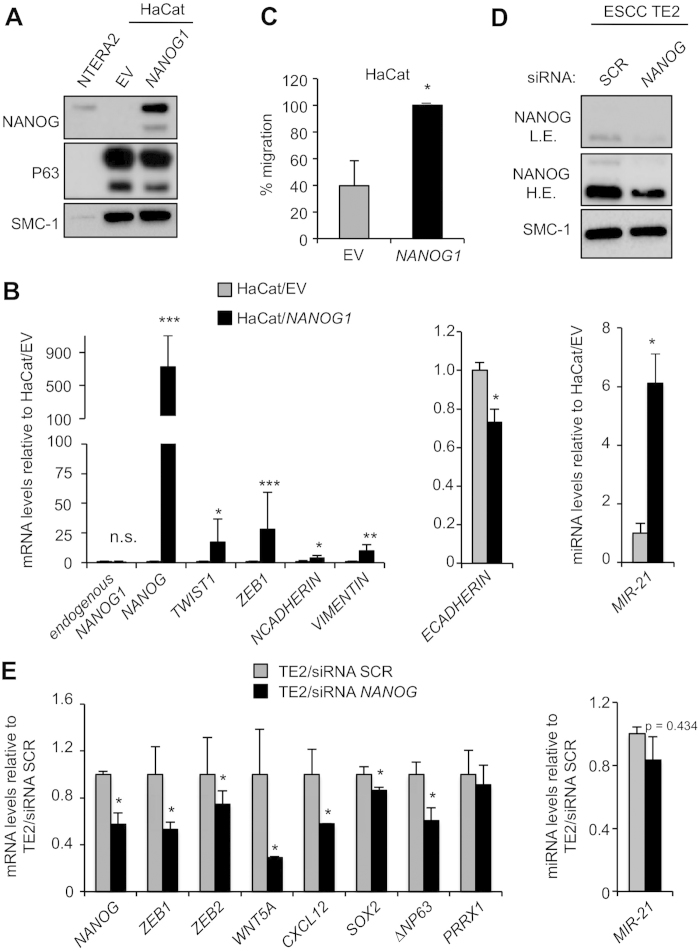
NANOG promotes EMT in epithelial cells . (**A**) Immunoblot of NANOG and P63 in nuclear extracts from HaCat cells transduced with a human NANOG expressing vector (HaCat/*NANOG1*) or empty vector control (HaCat/EV). NTERA2 teratocarcinoma cells were used as a positive control for NANOG, P63 was used as a keratinocyte marker, and SMC-1 was used as a loading control. (**B**) Relative mRNA levels of the indicated genes in HaCat cells treated as in (**A**). Samples were analyzed after puromycin selection. mRNA levels were normalized by *GAPDH* levels and then expressed as the ratios with respect to the levels in HaCat/EV (shown as fold change). Values correspond to two independent biological replicates (n = 2). (**C**) Percentage of migration of HaCat cells transduced with *NANOG1* or empty vector (n = 3). Areas were measured as percentage of migrated distance during a 24hr period (measured across the scratch wound width). (**D**) Immunoblots of NANOG using nuclear lysates of TE2 (human esophageal SCC cell line) cells transfected with pools of scrambled (siRNA SCR) or anti-NANOG (siRNA *NANOG*) siRNAs. Samples were analyzed 48 hr after transfection. SMC-1 was used as a loading control. L.E refers to low exposure of the film after incubation with ECL. H.E. refers to high exposure of the film after incubation with ECL. (**E**) Relative mRNA levels of the indicated genes in TE2 cells treated as in (**D**). mRNA levels were normalized by *GAPDH* levels and then expressed as the ratios with respect to the levels in TE2/siRNA SCR (shown as fold change). Values correspond to two transfections (n = 2). Bars in (**B**,**C**,**E**) correspond to mean ± SD. Statistical significance was determined by the two-tailed Student’s t test: n.s., non significant; (*) *P* < 0.05; (**) *P* < 0.01; (***) *P* < 0.001.

To investigate if NANOG overexpression in keratinocytes could recapitulate the observed *in vivo* phenotype, we infected human immortalized HaCat keratinocytes with a lentivirus encoding NANOG1 under the EF-1a promoter, as well as a control empty lentivirus (EV). After puromycin selection, we confirmed NANOG overexpression by immunoblotting (Fig. 5A), as well as by qRT-PCR (Fig. 5B). We confirmed by immunofluorescence that exogenous NANOG1 correctly localized to the nucleus (Supplementary Figure S5A). Additionally, we also confirmed upregulation of EMT mediators, such as *TWIST1*, *ZEB1*, *NCADHERIN, VIMENTIN* and *MIR-21*, as well as downregulation of *ECADHERIN* (Fig. 5B). Expression of *PRRX1* was below detection level in HaCat cells. To examine the functional consequences of NANOG overexpression, we focused on cell migration, which is a cell phenotype generally associated to EMT^33^. Interestingly, overexpression of NANOG in HaCat cells increased their migration capacity (Fig. 5C).

Finally, we silenced *NANOG* mRNA in TE2 ESCC cells using a siRNA-*NANOG* pool and compared it to its control (siRNA-scrambled or siRNA-SCR). We confirmed *NANOG* knockdown by immunoblotting (Fig. 5D) and by qRT-PCR (Fig. 5E). Interestingly, downregulation of *NANOG* led to decreased levels of EMT markers and genes related to stem features and malignancy (Fig. 5E). Also, shRNA-mediated downregulation of murine Nanog mRNA in spindle SCC MSC11A5 cells resulted in decreased migration activity (Supplementary Figure S5B).

Together, our results unravel a novel function of NANOG in promoting EMT and malignancy in squamous cell carcinomas.

## Discussion

Expression of high levels of NANOG is a distinctive feature of human squamous cell carcinomas (SCCs), such as head and neck SCCs and esophageal SCCs^12^,^13^,^15^,^16^. Here, we show that transgenic *Nanog* expression in the skin epidermis promotes the conversion of skin papillomas into skin SCCs. In particular, overexpression of *Nanog* greatly increased the rate of malignant conversion of papillomas into skin SCCs. Analysis of the global transcriptional profiles of papillomas by RNA-seq indicated that, at this early stage of tumorigenesis, transgenic NANOG is already upregulating key transcriptional regulators of EMT. A connection between NANOG and EMT had been already proposed based on studies with *in vitro* cultured cancer cells^34^,^35^. Here, we extend this concept to an *in vivo* model of tumorigenesis. In particular, *Nanog*-overexpressing papillomas show upregulation of several EMT mediators (*Zeb1*, *Zeb2, Twist*) and the master EMT inducer *Prrx1*, but not of the classical EMT inducers *Snail/Slug*. In this regard, it has been proposed that PRRX1 and SNAIL constitute independent pathways to induce EMT^36^. Also, *Nanog*-overexpressing papillomas upregulate *miR-21*, which is another key inducer of EMT^26^,^37^ previously shown to be regulated by NANOG in cultured cells^13^. Other miRNAs involved in the inhibition of EMT, such as *miR-200* and *miR-34*, were not found altered in *Nanog*-overexpressing papillomas. To further extend these findings, we show that NANOG regulates the expression of EMT mediators in primary mouse keratinocytes, in immortalized human keratinocytes (HaCat), and in human esophageal SCC cells (TE2). Moreover, we show in TE2 cells that endogenous NANOG directly binds to the promoter region of key EMT inducers and mediators, including, *PRRX1* and *ZEB2*. In agreement with the established role of NANOG in migration^9^,^10^, we show that NANOG overexpression in human keratinocyte-derived cells (HaCat) increases cell migration and, conversely, downregulation of NANOG in mouse SCC cells reduces their migration capacity.

In addition to EMT, we have observed that *Nanog*-overexpressing papillomas upregulate genes associated to cancer stem features, most notably, *Cxcl12/Sdf-1*^38^, *Lgr5*^39^ and *Pdgfra*^40^. Of note, endogenous NANOG binds the promoter of these genes in human esophageal SCC TE2 cells.

In summary, we demonstrate *in vivo* that NANOG has oncogenic potential to induce skin SCCs and this occurs in association with NANOG-dependent induction of an EMT program and cancer stem features.

## Methods

### Mice

Mice were housed at the specific pathogen-free (SPF) barrier area of the Spanish National Cancer Research Centre (CNIO), Madrid. Mice were observed on a daily basis and sacrificed when they showed overt signs of morbidity in accordance to the *Guidelines for Humane Endpoints for Animals Used in Biomedical Research* from the Council for International Organizations of Medical Sciences (CIOMS). All animal procedures were performed according to protocols approved by the CNIO-ISCIII Ethics Committee for Research and Animal Welfare (CEIyBA). The *tetO*-*Nanog* allele contains the *Nanog*-cDNA under the control of the doxycycline (DOX)-responsive promoter (*tetO*) inserted downstream of the *Col1a1* locus, (mice carrying this allele were a kind gift from Konrad Hochedlinger), and this allele is similar to the one described elsewhere for the generation of OCT4 inducible mice^17^. The *K5*-*rtTA* mouse contains a transgene with the reverse tetracycline transactivator (rtTA) cloned downstream from the bovine cytokeratin promoter (K5), as previously described^18^ (a kind gift from Silvio Gutkind). Mice carrying these alleles were combined and backcrossed (five consecutive backcrosses) with wild-type C57BL/6 mice to generate control *Col1a1*^*tetO-Nanog*/+^(CTR) and *Nanog*-inducible *Col1a1*^*tetO-Nanog*/+^; *K5-rTA*^tg/.^ (TG) littermate mice of C57BL/6 background. Transgene expression was induced in mice by replacing normal drinking water with 7.5% sucrose solution containing doxycycline (2 mg/ml) and the DOX-containing water was changed every 2-3 days.

To induce epidermal proliferation, mouse tail skin in telogen (resting) phase of the hair cycle was topically treated every 48 hr with TPA (20 nmol in acetone) (cat # P8139; Sigma) for a total of four doses. Control mice of each genotype were treated with acetone alone. Mice were sacrificed 24 hr after the last TPA treatment, and the tail skin was analyzed.

### DMBA/TPA chemical carcinogenesis assay

Age-matched (8–10 weeks old) mice of each genotype, CTR and TG were given doxycycline in their drinking water, which was changed every 2–3 days throughout the entire treatment. After 48 hr, a single dose of 100 nmol of DMBA (cat # D3254; Sigma) in 0.2 ml acetone was topically applied to the shaved dorsal skins. One week later, 17 nmol TPA (cat # P8139; Sigma) in 0.2 ml acetone was administered topically to the same area twice per week for 15 weeks. After 15 weeks of TPA treatment, mice were under observation for another period of 15 weeks and then sacrificed for histopathological analyses. Mice were evaluated weekly for papilloma development. Only tumors that had attained a size of 1 mm were counted. Mice with tumors larger than 1.5 cm were sacrificed considering humane endpoint before the end of the treatment, and analyzed histopathologically.

### Cell culture

Transformed human keratinocyte cell line HaCat, human teratocarcinoma cell line NTERA2, human esophageal SCC cell line TE2^41^ (kind gifts of Anil Rustgi), mouse squamous cell carcinoma cell lines MSC11B9^42^ and Pam212^43^, and mouse spindle cell carcinoma cell lines CarC^44^ and MSC11A5^42^ (kind gifts of Miguel Quintanilla) were all grown in (DMEM) (Gibco) supplemented with 10% fetal bovine serum (Invitrogen) and antibiotic-antimycotic solution (Gibco). Mouse transformed keratinocytes PDV^45^, mouse papilloma-derived cell line PB^46^, and mouse spindle cell carcinoma cell line CarB^44^ (kind gifts of Miguel Quintanilla) were grown Ham’s F-12 medium (Gibco) supplemented with 10% fetal bovine serum (Invitrogen) and antibiotic-antimycotic solution (Gibco). Mouse primary keratinocytes were freshly isolated from TG and CTR C57BL/6 neonates (days 1–3 post-partum). After dispase (STEMCELL Technologies) treatment the epidermis was separated from the dermis, minced and stirred. The derived cell suspension was then filtered through a sterile teflon mesh (Cell Strainer 0.7 μm, Falcon) to remove cornified sheets and keratinocytes were collected by centrifugation (160 × g) and seeded on collagen I pre-coated cell culture plates (BD Biosciences). Mouse primary keratinocytes were cultured in Cnt-07 (CELLnTEC) medium supplemented with antibiotic-antimycotic solution (Gibco). Transgene activation was done by adding doxycycline (DOX) to the medium (1 μg/ml).

### Histopathology, immunohistochemistry and in situ hybridization

Tissue samples were fixed in 10% buffered formalin, embedded in paraffin and sectioned at a thickness of 2.5 μm. Consecutive sections were stained with hematoxylin and eosin (H&E) and processed for immunohistochemistry performing antigen retrieval in a VENTANA DISCOVERY XT (Roche) with CC1 buffer and incubating them with the following two antibodies against mouse NANOG: Novus Biologicals, NB100-58842 lot# 5-1 (Fig. 1C,F **and S1**), and Cell Signaling Technology, D2A3, 8822 (**Figure S2E and S3A**). Other antibodies against mouse proteins were: p63 (Sigma, P3737), LORICRIN (Covance, PRB-145P), Ki67 (Master Diagnóstica, 000310QD), phospho-STAT3 (Tyr705) (Cell Signaling Technology, D23A7, 9145), E-CADHERIN (BD Biosciences, 610182), VIMENTIN (Cell Signaling Technology, D21H3, 5741) and phospho-AKT1 (S473) (Epitomis, EP2109Y, 2118-1). Following incubation with the primary antibodies, positive cells were visualized using 3,3-diaminobenzidine tetrahydrochloride plus (DAB+) as a chromogen. Counterstaining was performed with hematoxylin. Digital slides were acquired with a MIRAX SCAN (Zeiss) and images captured with the Pannoramic Viewer Software (3DHISTECH).

*In situ* hybridization (ISH) was performed with a *miR-21* Exiqon probe (microRNA ISH Optimization Kit 2 (FFPE)) and following the manufacturers’ instructions (Exiqon) using the Discovery XT immunohistochemistry system (Ventana Medical Systems).

### Immunofluorescence

Cells were plated in chambers and fixed in 4% paraformaldehyde for 10 min. Sections were blocked with 1% FBS for 1 hr at r.t., and then incubated for 2 hr at r.t. with an antibody recognizing the N-terminal region of human NANOG (Cell Signaling Technology, 4903) or the C-terminal region of human NANOG (R&D, AF1997) in Dako Antibody Diluent with Background Reducing Components. After washing (3 times with PBS 0.1% Triton X-100), slides were then stained with donkey anti-goat AlexaFluor555-conjugated secondary antibody for the C-terminal NANOG antibody or with goat anti-rabbit AlexaFluor488-conjugated secondary antibody for the N-terminal NANOG antibody (both secondary antibodies from Invitrogen) in Dako REAL^TM^ buffer, 1 hr at r.t., followed by 4’,6-Diamidino-2-phenylindole (DAPI) staining to visualize the nuclei. Slides were mounted with Vectashield antifade medium (Vector Laboratories) before confocal analysis. Confocal microscopy was performed with a TCS SP5 laser scanning spectral microscope (Leica Microsystems) equipped with a Plan-Apochromat 40x/1.2 NA oil objective. 8-bits images were acquired using the Leica LAS AF v.2.1 software (Leica Microsystems). The pictures show the maximum projection of Z-stacks.

### RNA sequencing

The RNA-seq data has been deposited in the GEO repository (accession number GSE56566). Total RNA was extracted from papillomas of 4 different CTR mice and papillomas of 3 different TG mice collected at the end of the protocol or at the humane endpoint using RNeasy Fibrous Tissue Mini Kit (Qiagen). 1 μg of total RNA, with RIN (RNA integrity number) numbers above 7 (Agilent 2100 Bioanalyzer), were used. PolyA+fractions were processed using TruSeq Stranded mRNA Sample Preparation Kit (Illumina). The resulting directional cDNA libraries were sequenced for 40 bases in a single-read format (Genome Analyzer IIx, Illumina). Reads were aligned to the mouse genome (GRCm38/mm10) with TopHat-2.0.4^47^ (using Bowtie 0.12.7^48^ and Samtools 0.1.16^49^; allowing two mismatches and five multihits. Transcripts assembly, estimation of their abundances and differential expression were calculated with Cufflinks 1.3.0^47^, using the mouse genome annotation data set GRCm38/mm10 from the UCSC Genome Browser^50^.

GSEAPreranked was used to perform a gene set enrichment analysis of the published EMT and CSC pathways. We used the RNA-seq gene list ranked by statistic, setting ‘gene set’ as the permutation method and we run it with 1000 permutations. We considered only those gene sets with significant enrichment levels (FDR q-value < 0.05)^51^.

### Quantitative real-time PCR

In the case of cultured cells and tissues, total RNA was obtained using RNeasy Mini Kit (Qiagen) following the manufacturer’s instructions. In the case of miRNA analysis, total RNA was obtained from tissues using the miRNeasy Mini Kit (Qiagen) following the manufacturer’s instructions. To generate cDNA, total RNA was reverse transcribed using iScript Advanced First Strand cDNA synthesis kit (BioRad), according to the manufacturer’s protocols. Quantitative real-time PCR (qRT-PCR) was performed using *GoTaq qPCR* Master Mix (Promega) in an ABI PRISM 7700 thermocycler (Applied Biosystems). Calculation for the values was according to the ∆∆Ct method^52^ and using *Gapdh/GAPDH* as control.

TaqMan® MicroRNA Assays (Applied Biosystems) were used to quantify miRNAs in samples according to the manufacturer instructions with the TaqMan® Universal PCR Master Mix reagent kit (Applied Biosystems). Normalization was performed using U6 as housekeeping small RNA. qRT-PCR for miRNA was performed in an ABI 7500fast Real-Time PCR System.

### Protein analysis

Nuclear protein extracts were prepared using the Nuclear/Cytosol Fractionation kit (BioVision) following protocols provided by the manufacturer. Total lysates were prepared using lysis buffer (50 mM Tris-HCl pH 7.5, 150 mM NaCl, 4 mM CaCl, 1.5% Triton X-100, protease inhibitors and micrococcal nuclease). In the case of tissue extracts, lysates were homogenized using a Precellys 24 tissue homogenizer. For immunoblotting, protein extracts were resolved using NuPAGE 4–12% gradient Bis-Tris gels, transferred to nitrocellulose and incubated with the corresponding antibodies. For murine NANOG detection, we used Calbiochem, SC1000. For human NANOG detection, we used Cell Signaling, D73G4XP. Other antibodies used were: γ-TUBULIN (Sigma, GTU-88), phospho-ERK1/2 (Thr202/Tyr204) (Cell Signaling, 9101), total ERK1/2 (Cell Signaling, 9102), phospho-AKT (Ser473) (Cell Signaling, 4058), p63 (Novus Biologicals, 4A4, NB100-691) and SMC1 (Bethyl, A300-055A). Quantification of immunoblots was done using the ImageJ software. Uncropped images of the immunoblots are shown in Supplementary Figure S6.

### Chromatin immunoprecipitation

Cells were crosslinked with 1% formaldehyde for 15 min at r.t. Crosslinking was stopped by the addition of glycine to a final concentration of 0.125 M. Fixed cells were lysed in lysis buffer (1% SDS, 10 mM EDTA, 50 mM Tris-HCl, pH 8.0 and protease inhibitors) and sonicated. As input, 80 μg of protein extract were reserved. For immunoprecipitation, 800 μg of protein were diluted in dilution buffer (1.1% Triton X-100, 0.01% SDS, 1.2 mM EDTA, 167 mM NaCl and 16.7mM Tris-HCl, pH 8.0 and protease inhibitors), pre-cleared with A/G plus-agarose pre-blocked with BSA (Santa Cruz) and incubated with an antibody against human NANOG (R&D Biosystems AF1997) or goat IgG isotype (Jackson ImmunoResearch, 005-000-003). Immune complexes were precipitated with A/G plus-agarose pre-blocked with BSA and washed sequentially with low-salt immune complex wash buffer (0.1% SDS, 1% Triton X-100, 2 mM EDTA, 150 mM NaCl, 20 mM Tris-HCl pH 8.0), high-salt immune complex wash buffer (0.1% SDS, 1% Triton X-100, 2 mM EDTA, 500 mM NaCl, 20 mM Tris-HCl, pH 8.0), LiCl immune complex wash buffer (0.25 M LiCl, 1% NP-40, 1% deoxycholate-Na, 1 mM EDTA, 10 mM Tris-HCl, pH 8.0), and TE buffer (1 mM EDTA, 10 mM Tris-HCl pH 8.0), and finally eluted in elution buffer (1% SDS, 50 mM NaHCO_3_). All samples, including inputs, were subjected to reverse crosslinking, treated with proteinase K and DNase-free RNase, and DNA was extracted with QIAGEN PCR purification column (Qiagen) and resuspended in TE buffer. qPCR was performed in triplicate (technical replicates) in two independent experiments. Negative control corresponds to a genomic region that does not bind NANOG^53^. As a positive control, we have used the NANOG proximal promoter region, since NANOG is known to bind to its own promoter^53^.

### Cell transfection

For *NANOG* silencing, TE2 human esophageal cancer cells were transfected with a pool of four siRNA duplexes targeting *NANOG* (ON-TARGETplus Human NANOG (79923) siRNA, SMARTpool, Dharmacon) or with a pool of scramble siRNA duplexes (ON-TARGET*plus* Non-targeting Control Pool, SMARTpool, Dharmacon) using Lipofectamine RNAiMAX in Opti-MEM I (Invitrogen) according to the manufacturer’s instructions. Cells were processed for RNA extraction 48 hr after transfection.

For mouse *Nanog* silencing in MSC11A5 cells, lentiviruses containing pLKO.1-scramble shRNA (shSCR) and pLKO.1-Nanog shRNA (sh*Nanog*) (5′-GCCAACCTGTACTATGTTTAA-3′)^54^, were produced in 293T cells using the packaging plasmids pLP1, pLP2 and pLP/VSVG (Invitrogen), by cotransfecting all plasmids using FuGENE-6 (Promega) according to the manufacturer’s protocol. Cells were plated the day prior to infection, and supernatans were collected every 12 hours for two days from 293T cells. Cells were selected with puromycin to obtain stable sh*Nanog* cell lines.

For NANOG overexpression, HaCat cells were infected with lentiviruses expressing NANOG1 pSin-EF2-Nanog-Pur (Addgene plasmid #16578)^55^ and with control empty vector pSin-EF2-empty-Pur (produced by excising *NANOG1* by BamHI restriction and re-ligation). Lentiviruses were produced in 293T cells using the lentivirus packaging plasmids pLP1, pLP2 and pLP/VSVG (Invitrogen), by cotransfecting all plasmids using Fugene HD transfection reagent (Promega) according to the manufacturer’s protocol. Cells were plated the day prior to infection, and supernatants were collected 48 hr from 293T cells after transfection. HaCat cells were infected with the lentiviruses and were selected with puromycin to obtain stable HaCat/NANOG1 or HaCat/EV cells.

### *In vitro* migration assay

For the *in vitro* wounding assay, serum starved cells were pretreated with 10 μg/ml mitomycin C for 2 hr to block proliferation and a cell-free area was created by scratching the monolayer with a 200 μl pipette tip. Cell migration into the wound area was monitored in serum-free medium or in the presence of 10% FBS. Photographs were taken using a phase-contrast microscope (DIAPHOT 300; Nikon). Migration into the denuded area was monitored for up to 24 hr. Quantification of migration was done using the ImageJ software.

### Statistical analysis

Values were expressed as mean ± SD and differences with *P* value < 0.05 were considered significant ((*) *P* < 0.05, (**) *P* < 0.01, (***) *P* < 0.001). Comparisons between two groups were performed using unpaired two-tailed Student’s *t*-test. For comparing ratios of tumor incidence (Fig. 2E) we used the Fisher´s exact test. These statistical analyses were performed using GraphPad Prism software.

## Author Contributions

A.R.P. performed most of the experiments, contributed to the experimental design, data analysis, discussion and writing the paper; D.P. performed some of the experiments, contributed to the experimental design, data analysis, discussion and writing the paper; N.A. performed some experiments and contributed to writing of the paper; M.C. performed most of the histological analyses and contributed to data analysis; O.G. and G.G.-L. performed the biocomputing analyses; O.D. performed the RNAseq; M.D. performed the miRNA analyses; J.M.P. contributed to the interpretation of data, discussion, and writing; M.S. designed and supervised the study, secured funding, analyzed the data, and wrote the manuscript. All authors discussed the results and commented on the manuscript.

## Additional Information

**How to cite this article**: Palla, A. R. *et al.* The pluripotency factor NANOG promotes the formation of squamous cell carcinomas. *Sci. Rep.* **5**, 10205; doi: 10.1038/srep10205 (2015).

## Supplementary Material

Supplementary Information
