# Supplementary material for: The pluripotency factor NANOG promotes the formation of squamous cell carcinomas
Source: Sci Rep. 2015 May 19;5:10205. doi: 10.1038/srep10205 (PMC4437308; doi:10.1038/srep10205)
Supplement: Supplementary Information [file srep10205-s1.pdf]

SUPPLEMENTAL DATA

The pluripotency factor NANOG promotes the formation of squamous cell carcinomas

Adelaida R. Palla, Daniela Piazzolla, Noelia Alcazar, Marta Cañamero, Osvaldo Graña, Gonzalo Gómez-López, Orlando Dominguez, Marta Dueñas, Jesús M. Paramio and Manuel Serrano

SUPPLEMENTAL DATA

Figure S1
Figure S2
Figure S3
Figure S4
Figure S5
Table S1
SUPPLEMENTARY METHODS

Supplementary Figure S1. Palla *et al.*, submitted

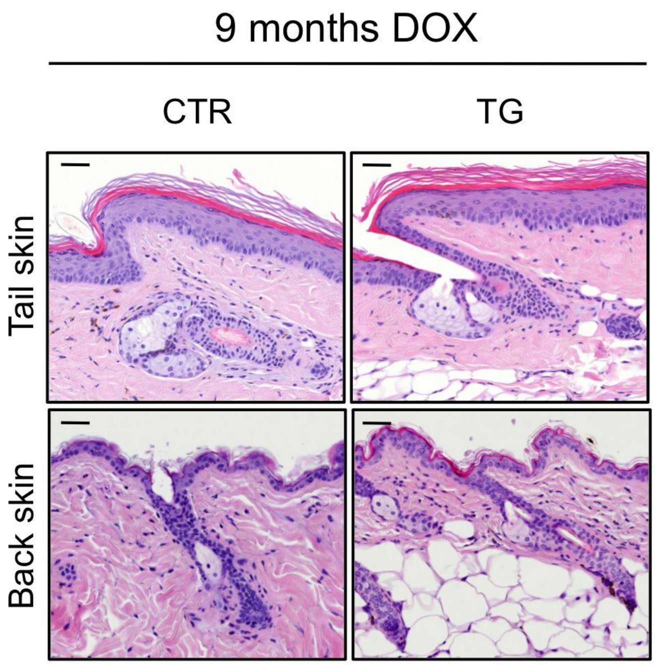

Figure S1. 9-month continuous NANOG overexpression does not lead to epidermal alterations. Representative hematoxylin and eosin (H&E) staining of CTR and TG mice after 9 month-doxycycline treatment of tail skin and back skin (bars correspond to 50 μm).

Supplementary Figure S2. Palla *et al.*, submitted

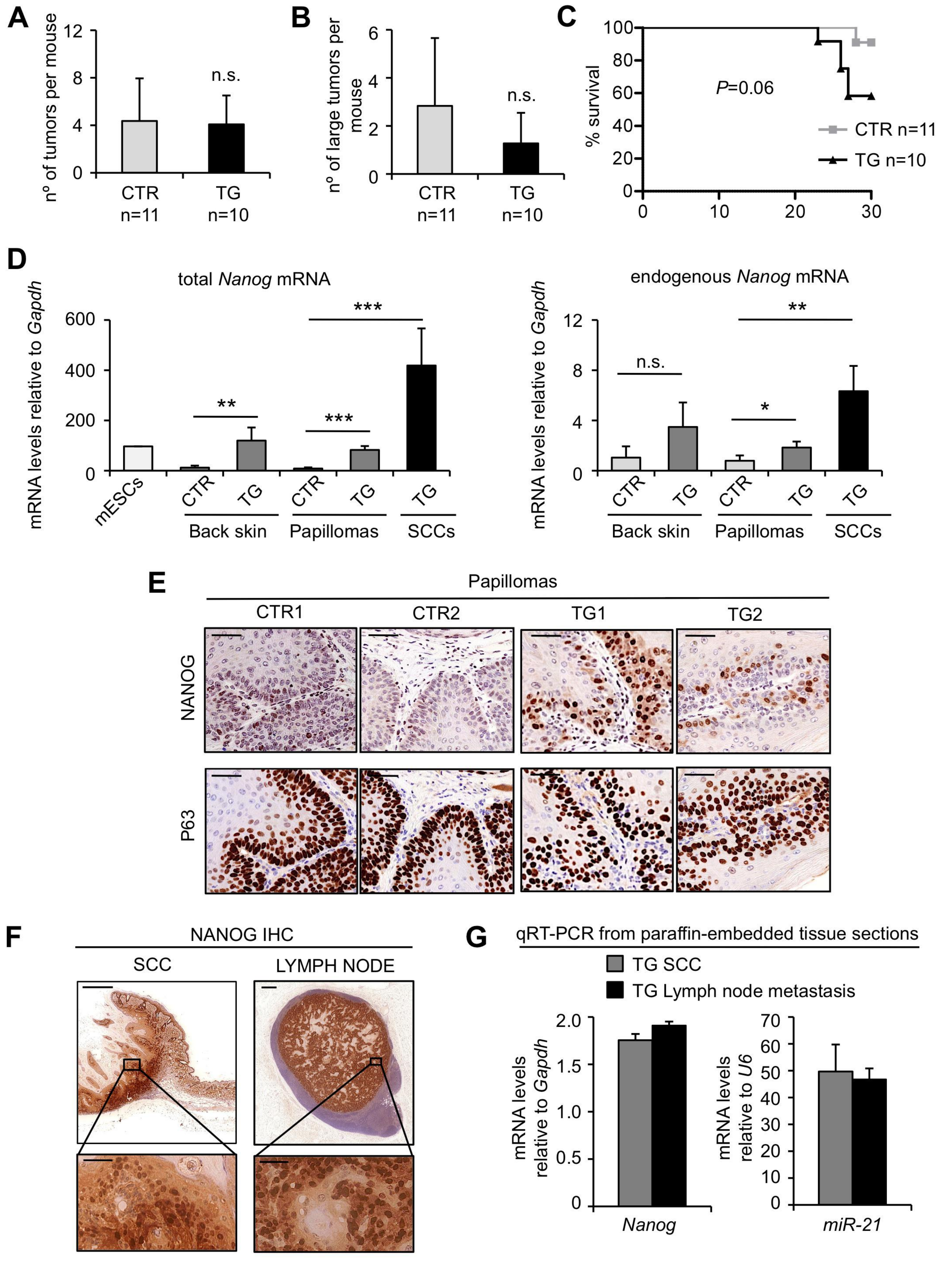

Figure S2. NANOG overexpression leads to squamous cell carcinoma formation upon DMBA/TPA chemical carcinogenesis.

- **(A)** and **(B)** Average number of total tumors (A) and large tumors (>4mm largest section) (B) per mouse. Quantification was performed at the humane endpoint or at the end of the experiment (30 weeks).
- **(C)** Survival curve of CTR and TG mice upon DMBA/TPA treatment and continuous treatment with doxycycline. Statistical significance was determined by the Long-rank (Mantel-Cox) test. (n), number of mice.
- **(D)** Relative total *Nanog* mRNA levels (left) and endogenous *Nanog* mRNA levels (right) in CTR (n=4) and TG (n=4) back skin; CTR (n=4) and TG (n=3) papillomas; and TG (n=3) SCCs. Each sample (back skin, papilloma or SCC) is from a different mouse at the humane endpoint or at the end of the experiment (30 weeks). mRNA levels were normalized by *Gapdh* levels. As a positive control, mouse embryonic stem cells (mESCs) were used.
- **(E)** IHC of NANOG and P63 in representative CTR and TG papillomas. P63 was used as a marker of epidermal progenitor cells.
- **(F)** IHC of NANOG in a SCC and lymph node metastasis from the same mouse. (Bars correspond to 500µm in the low magnification pictures and to 50µm in the high magnification pictures).
- **(G)** mRNA levels in SCC and lymph node metastasis from paraffin embedded tissue sections of the indicated genes.

Bars in graphs correspond to mean ± SD of the indicated number of mice (n). Statistical significance was determined by the two-tailed Student's t test: n.s. non significant; (*) $P<0.05$; (**) $P<0.01$; (***) $P<0.001$.

Supplementary Figure S3. Palla *et al.*, submitted

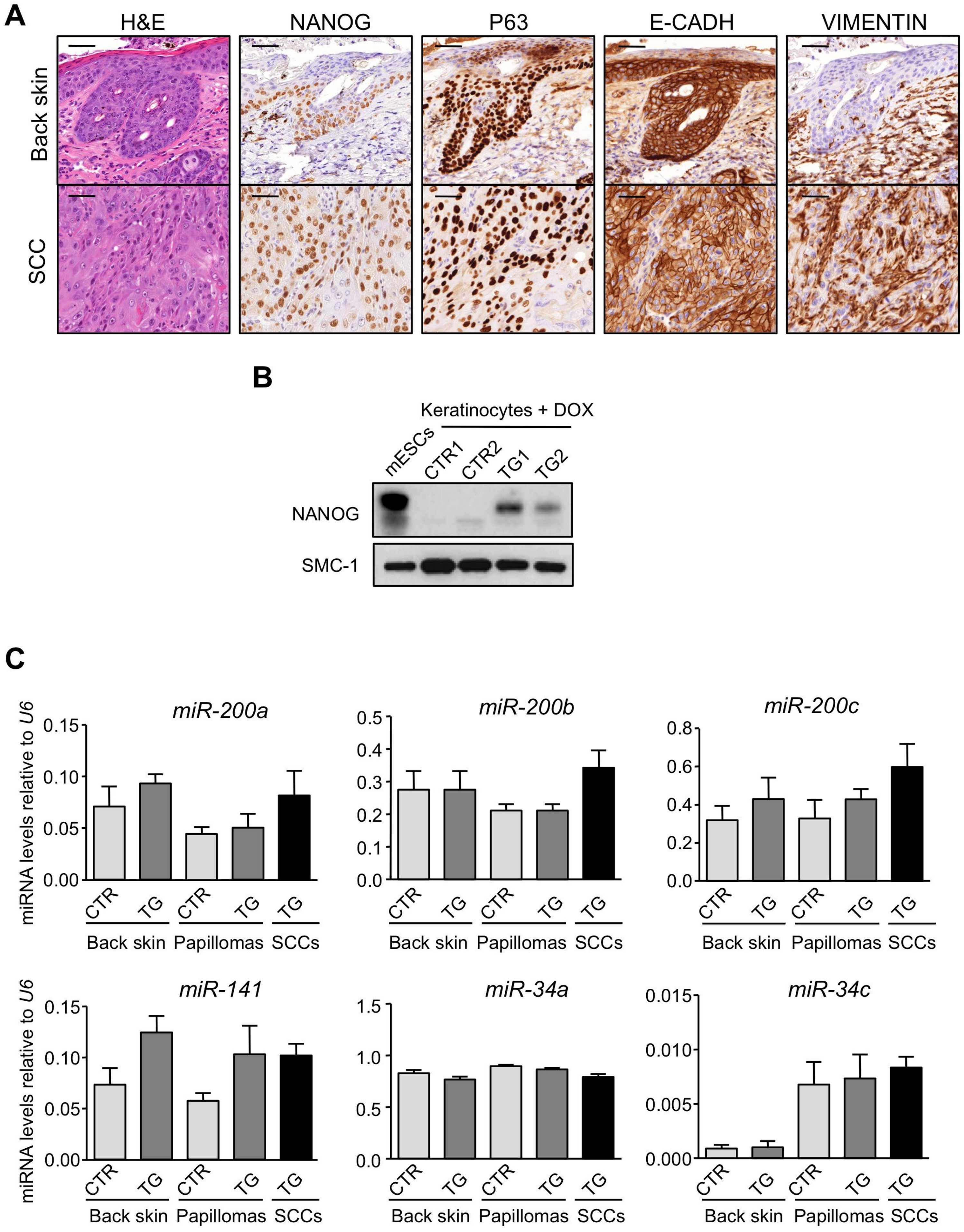

Figure S3. Overexpression of NANOG promotes EMT in tumors and epithelial cells.

(A) Representative hematoxylin and eosin (H&E) and IHC for NANOG, P63, E-CADHERIN and VIMENTIN staining of serial sections of a TG SCC. P63 was used as a marker of epidermal progenitor cells. Top corresponds to magnified back skin and bottom to magnified invasive carcinoma (bars correspond to 50 μm).

(B) Immunoblots of NANOG using nuclear lysates from CTR and TG keratinocytes after 48 hr doxycycline treatment. SMC-1 was used as a loading control. Mouse embryonic stem cells (mESCs) were used as a positive control.

(C) MicroRNA expression levels relative to *U6* in CTR (n=4) and TG (n=4) back skin; CTR (n=4) and TG (n=3) papillomas; and TG (n=3) SCCs. (n), number of samples, each from a different mouse after 30 weeks of doxycycline treatment. No statistically significant differences were found using the two-tailed Student's t test.

Supplementary Figure S4. Palla *et al.*, submitted

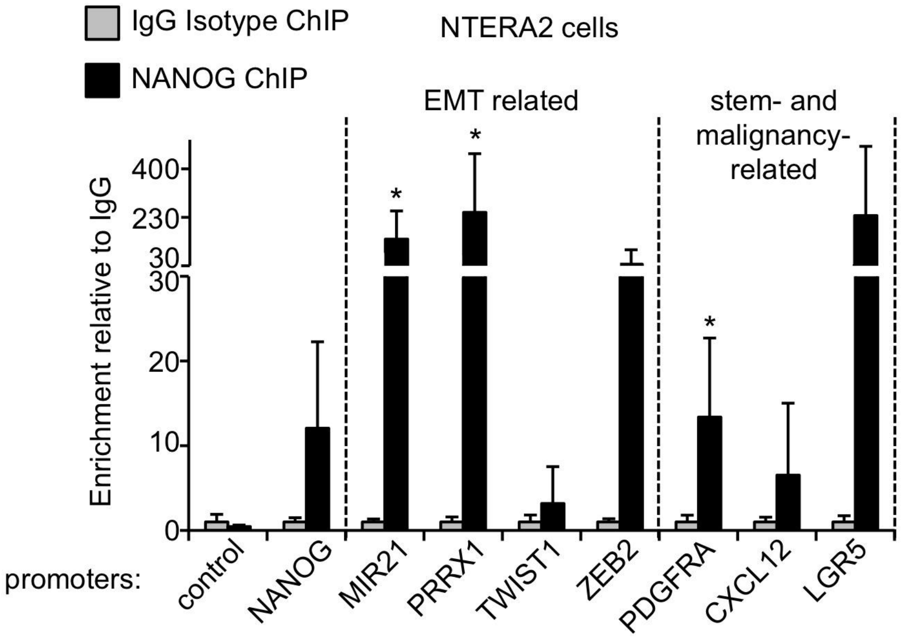

Figure S4. Chromatin immunoprecipitation (ChIP) of NANOG and goat IgG in NTERA2 teratocarcinoma cells. Values were first normalized to the input values, and then compared to levels of the IgG control (shown as fold change). Values correspond to mean ± SD of two independent biological replicates (n=2). Control, genomic region that does not bind NANOG (see Supplementary Methods). Statistical significance was determined by the two-tailed Student's t test: (*) $P<0.05$. (**) $P<0.01$.

Supplementary Figure S5. Palla *et al.*, submitted

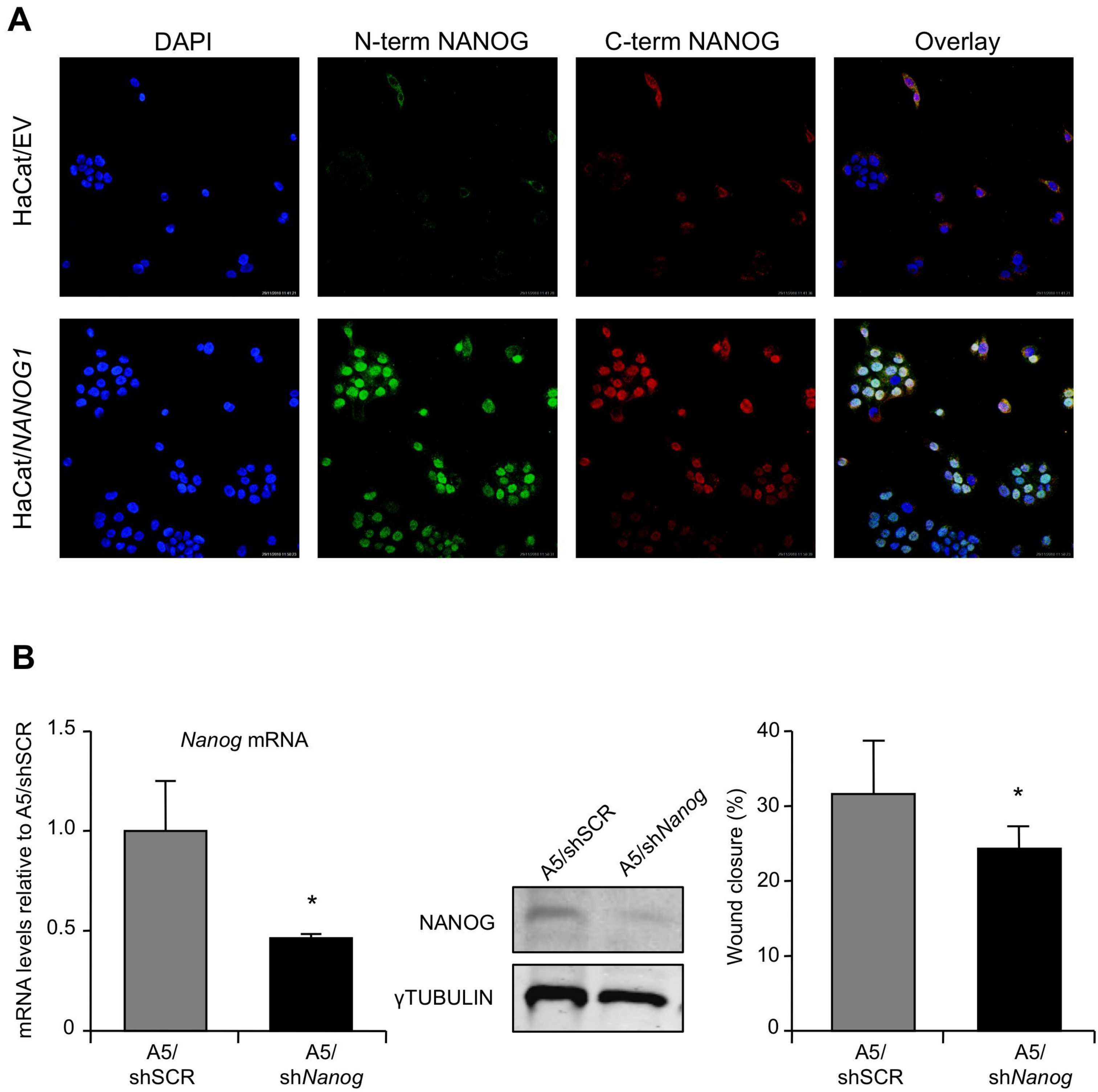

Figure S5.

(A) Immunofluorescence of NANOG using two different antibodies (C-terminal and N-terminal) in HaCat cells infected with a human NANOG expressing vector or empty vector control (EV). Samples were analyzed after puromycin selection. DAPI was used to view the cell nuclei.

(B) Left: Relative *Nanog* mRNA levels in MSC11A5 cells transfected with shSCR (control) or sh*Nanog* after puromycin selection. mRNA levels were normalized by *Gapdh* levels and then, compared to levels of A5/shSCR (shown as fold change) (n=3). Middle: Immunoblots of NANOG using total lysates from MSC11A5 cells treated as in (B). γTUBULIN is used as a loading control. Right: Percentage of wound closure at 6hr of MSC11A5 cells treated as in (B). Areas were measured as percentage of migrated distance (measured across the scratch wound width) (n=8).

Bars correspond to mean ± SD of one experiment. Statistical significance was determined by the two-tailed Student's t test: (*) $P<0.05$.

Supplementary Figure S6. Palla *et al.*, submitted

Figure 2F

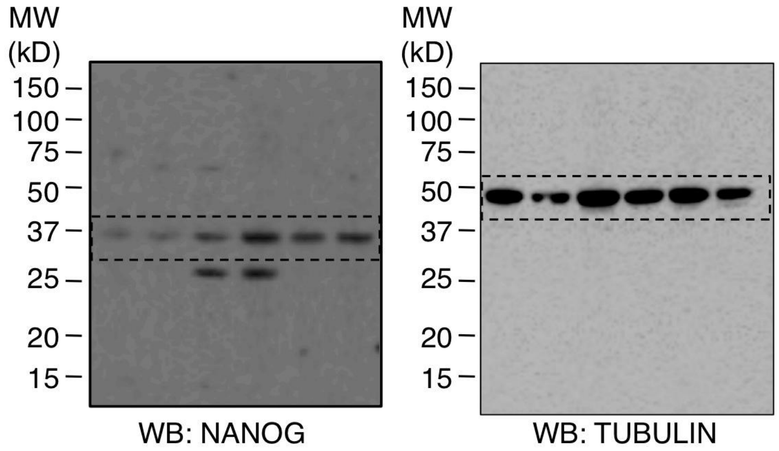

Figure 3G

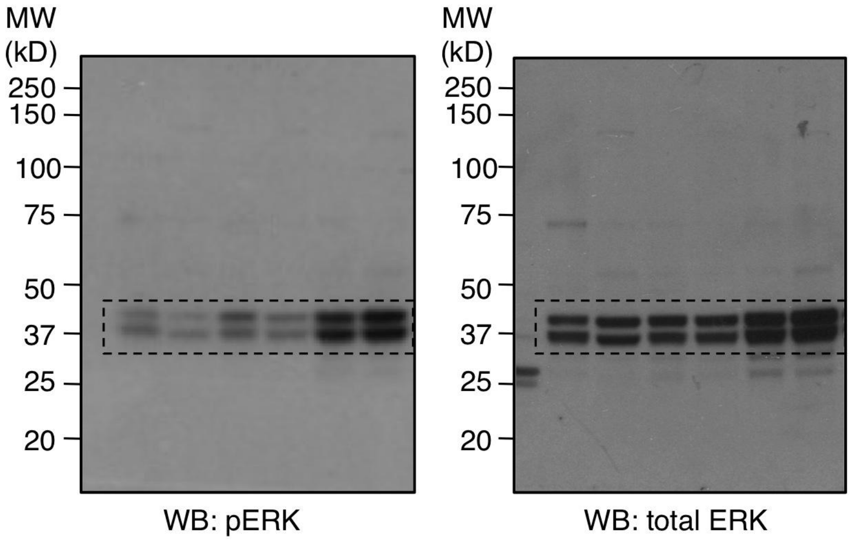

Figure 5A

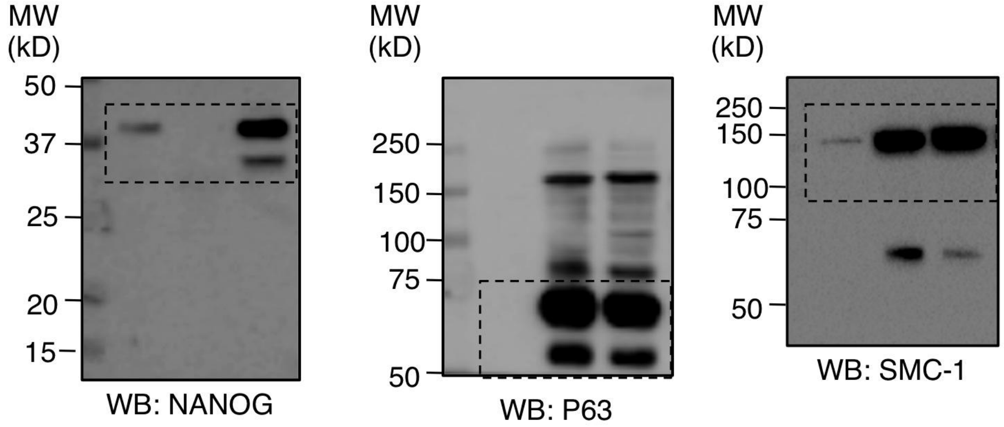

Supplementary Figure S6. (continued) Palla *et al.*, submitted

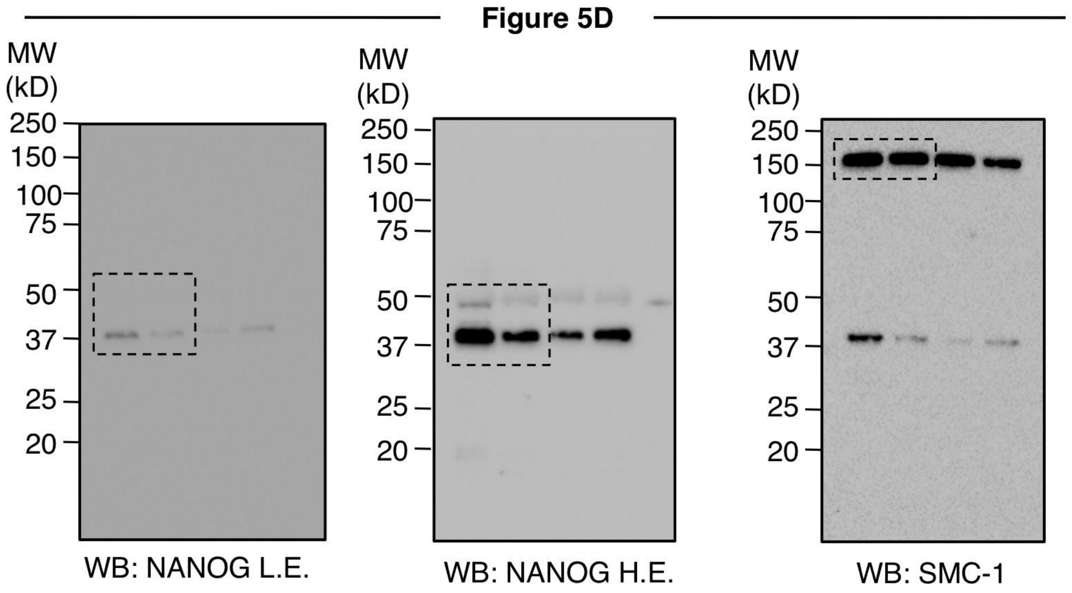

Figure S6. Uncropped images of the blots shown in Figs 2F, 3G, 5A and 5D. MW, molecular weight in kilodalton (kD); L.E, low exposure; H.E., high exposure

Supplementary Table S1. Upregulated differentially expressed genes (DEG) in NANOG-overexpressing papillomas versus controls (Orange: EMT-related genes; Blue: cancer stem-related genes; Green: EMT and cancer stem-related genes)

DEG (FDR<0.05)[a]

Upregulated in TG (n=3) vs CTR (n=4) papillomas

Gene	Log2 (fold change)	NOM p-val	FDR q-val
Mybpc1	>10	3.94E-04	4.27E-02
Myh4	9.77	3.87E-10	6.18E-07
Xirp2	8.84	8.05E-08	3.60E-05
Actn3	7.91	9.19E-06	2.44E-03
Tnnc2	7.86	1.67E-06	5.47E-04
Ttn	7.83	4.44E-16	2.48E-12
Cmya5	7.71	9.43E-08	4.06E-05
Car3	7.56	1.46E-11	4.92E-08
Ppp1r3a	7.46	7.86E-06	2.14E-03
Myl1	7.50	1.82E-08	1.13E-05
Myh8	6.86	3.32E-05	6.51E-03
Myh2	6.83	2.99E-09	2.87E-06
Ckm	6.69	1.76E-11	4.92E-08
Mybpc2	6.16	5.92E-08	2.99E-05
Atp2a1	6.56	1.82E-08	1.13E-05
Nrap	6.52	4.95E-04	4.99E-02
Tspan1	6.34	1.34E-08	8.83E-06
Cyp2g1	6.26	7.85E-05	1.33E-02
Sgcg	6.21	2.82E-04	3.32E-02
Myh1	6.20	2.97E-11	6.65E-08

Myom2	6.01	2.35E-04	2.96E-02
Casq1	5.88	8.68E-05	1.40E-02
Thbs4	5.84	1.63E-05	3.64E-03
Slc15a2	5.59	5.62E-06	1.57E-03
Pygm	5.48	3.16E-09	2.872E-06
Muc4	5.43	4.66E-08	2.60E-05
Myoz1	5.24	1.36E-04	1.97E-02
Ldb3	5.20	9.29E-09	6.49E-06
H19	5.15	3.33E-09	2.87E-06
Mb	5.11	6.29E-05	1.100E-02
Rps4y2	4.83	3.13E-10	5.83E-07
Neb	4.79	1.05E-09	1.31E-06
Hand1	4.77	3.03E-06	8.93E-04
Krt71	4.64	1.99E-04	2.75E-02
Scara5	4.58	2.21E-04	2.88E-02
Ppbp	4.46	6.57E-08	3.06E-05
Tcap	4.37	4.34E-04	4.65E-02
Dynap	4.28	2.91E-07	1.084E-04
Nov	4.20	4.12E-05	7.83E-03
Gm9573	4.18	6.15E-08	2.99E-05
Fa2h	4.06	2.66E-04	3.20E-02
Cxcl5	4.01	7.31E-09	5.45E-06
Pkia	3.98	3.00E-04	3.49E-02
Sell	3.91	1.07E-04	1.65E-02
Atp1a2	3.85	9.05E-05	1.45E-02
Des	3.83	2.43E-06	7.73E-04
Tmc5	3.78	3.85E-04	4.22E-02

Myom1	3.72	2.92E-05	5.83E-03
C3	3.62	1.46E-05	3.33E-03
Mylpf	3.60	1.19E-06	4.02E-04
Cxcl12/Sdf-1	3.48	8.76E-07	3.06E-04
Acta1	3.43	4.84E-07	1.75E-04
Slc6a14	3.39	5.37E-08	2.86E-05
Col14a1	3.38	4.04E-05	7.80E-03
Retnla	3.37	3.34E-04	3.85E-02
Clec4e	3.36	1.15E-04	1.72E-02
Nanog	3.35	2.90E-09	2.87E-06
Cd300lf	3.30	2.52E-04	3.07E-02
Fhl1	3.27	5.39E-09	4.31E-06
Ltf	3.26	2.71E-04	3.23E-02
Plet1	3.24	1.45E-07	5.99E-05
Irg1	3.23	4.80E-05	8.95E-03
Slfn4	3.16	2.49E-06	7.74E-04
Ryr1	3.15	8.07E-05	1.35E-02
Gprc5a	2.99	2.27E-05	4.90E-03
Cpn1	2.96	2.36E-04	2.96E-02
Tgm2	2.92	5.20E-05	9.53E-03
Cilp	2.90	8.34E-05	1.37E-02
Cxcl3	2.83	1.15E-05	2.70E-03
Scn8a	2.82	1.13E-05	2.69E-03
Hdc	2.72	4.67E-04	4.83E-02
Nmes1	2.58	6.08E-05	1.09E-02
Jph2	2.56	1.47E-04	2.11E-02
Stfa2l1	2.52	2.65E-06	7.99E-04

Stfa2	2.51	2.27E-05	4.89E-03
Lpo	2.41	4.85072E-04	4.93E-02
Ivl	2.38	2.42E-05	5.01E-03
Lcn2	2.36	1.05E-04	1.63E-02
Il1b	2.24	3.61E-04	4.04E-02
Plat	2.21	1.10E-04	1.67E-02
Gm5416	2.21	2.48E-04	3.05E-02
Srgn	2.15	3.76E-04	4.16E-02
Aldh1a3	2.13	2.64E-05	5.36E-03
Slc5a8	2.13	1.06E-05	2.61E-03
Sec14l2	2.09	2.1E-04	2.84E-02
Tdh	1.80	4.77E-04	4.89E-02
Lgals1	1.77	3.44E-04	3.88E-02
Lcp1	1.76	1.58E-04	2.20E-02
Prnd	1.70	1.07E-05	2.61E-03
Plau	1.59	3.37E-04	3.85E-02
Prrx1	1.39	4.44E-04	4.68E-02
Acpp	1.35	1.02E-05	2.61E-03
Wnt5a	1.32	4.49E-04	4.69E-02
Slco3a1	1.13	2.2E-04	2.89E-02
Vcan	1.02	2.32E-04	2.96E-02
Liph	0.82	1.88E-07	7.51E-05
Hdac7	0.51	2.89E-08	1.07E-05

[a] FDR q-val, false discovery rate q-value; NOM p-val, nominal p-value

Supplementary Methods

Quantitative real-time PCR

PCR primers for mouse transcripts were as follows:

Primer		Sequence 5’ → 3'
total mNanog	fw	CAAGGGTCTGCTACTGAGATGCTCTG
	rev	TTTTGTTTGGGACTGGTAGAAGAATCAG
endogenous mNanog	fw	CTTCCCTCGCCATCACACT
	rev	ACAGTCCGCATCTTCTGCTTC
mPrrx1	fw	TTTGAGCGGACACATTACCC
	rev	GCTCATTCCTGCGGAACTT
mTwist1	fw	ACGCTGCCCTCGGACAA
	rev	CCTGGCCGCCAGTTTG
mZeb1	fw	GCTGGCAAGACAACGTGAAAG
	rev	GCCTCAGGATAAATGACGGC
mZeb2	fw	CAGGCTCGGAGACAGATGAAG
	rev	CTTGCAGAATCTCGCCACTG
mE-cadherin	fw	AGCTTGTGGAGCTTTAGATGC
	rev	TTTTCGGAAGACTCCCGATTCA
mKrt18	fw	GCACTCAGGAGAAGGAGCAG
	rev	CAACAGGCTCCACTTGGTCT
mN-cadherin	fw	CTGATAGCCCGGTTTCACTTG
	rev	CAGGCTTTGATCCCTCTGGA
mVimentin	fw	CCACTTTCCGTTCAAGGTCAAG
	rev	CGGCTGCGAGAGAAATTGC
mVcan	fw	AACTGCTTTCCTGATTGGCA
	rev	GATAACAGGTGCCTCCGTTG
mCxcl12	fw	TGTGGCTGCTCATCCTCTTTA
	rev	ACAACCTGGTCCAACTCAGC
mH19	fw	GAACAGAAGCATTCTAGGCTGG
	rev	TTCTAAGTGAATTACGGTGGGTG
mLrg1	fw	CCATGTCAGTGTGCAGATTC
	rev	AAGAGTGAGAGGTGGAAGAG
mPecam1	fw	CGTGTGGACCAACCTTACG
	rev	AAGCCATTCTTGCACAGCTC
mPdgfra	fw	GCCAGACATTGACCCTGTTC
	rev	TCTCGATGGCACTCTCTTCC
mSrc2	fw	ACATCCTGGTGGGGGAATAC
	rev	CTGTCCACTTGATGGGGAAC
mWnt5a	fw	TGTCTTTGGCAGGGTGATG
	rev	AGCCACAGGTAGACAGCTCG
mGapdh	fw	TTCACCACCATGGAGAAGGC
	rev	CCCTTTTGGCTCCACCCT

PCR primers for human transcripts were as follows:

Primer		Sequence 5’ → 3'
total hNANOG	fw	CACTTCTGCAGAGAAGAGTGTCG
	rev	AGCTGGGTGGAAGAGAACACAG
endogenous hNANOG1	fw	TTCATTATAAATCTAGAGACTCCAGGA
	rev	CTTTGGGACTGGTGGAAGAATC
hSNAIL	fw	CGAGCTGCAGGACTCTAAT
	rev	CCACTGTCCTCATCTGACA
hTWIST1	fw	CCGGAGACCTAGATGTCATTG
	rev	CACGCCCTGTTTCTTTGAAT
hZEB1	fw	GATGATGAATGCGAGTCAGATGC
	rev	ACAGCAGTGTCTTGTTGTTGT
hZEB2	fw	CAAGAGGCGCAAACAAGCC
	rev	GGTTGGCAATACCGTCATCC
hE-CADHERIN	fw	CGAGAGCTACACGTTCACGG
	rev	GTGTCGAGGGAAAAATAGGCTG
hN-CADHERIN	fw	GAGGAGTCAGTGAAGGAGTCA
	rev	GGCAAGTTGATTGGAGGGATG
hVIMENTIN	fw	AGTCCACTGAGTACCGGAGAC
	rev	GGTTCCTTTAAGGGCATCCAC
hCXCL12	fw	GAGCCAACGTCAAGCATCTC
	rev	TCCACTTTAGCTTCGGGTCA
hLGR5	fw	AACATCAGTCAGCTGCTCCC
	rev	TGTAAAGGCCAGTGAATGCTC
hΔNp63	fw	CAGACTCAATTTAGTGAGCC
	rev	CTGCTGGTCCATGCTGTT
hSOX2	fw	GGGAAATGGGAGGGGTGCAAAAGAGG
	rev	TTGCGTGAGTGTGGATGGGATTGGTG
hWNT5A	fw	AGGTGGAACTGCAGCACTGT
	rev	CTCATGGCGTTCACCACC
hGAPDH	fw	AGCCACATCGCTCAGACACC
	rev	GTACTCAGCGGCCAGCATGG

Chromatin immunoprecipitation

Primers for PCR after ChIP were as follows in the proximal promoter regions of the indicated genes:

Primer		Sequence 5’ → 3'
Negative control	fw	TTGTGTAGCACCAGGGTCA
	rev	GCCTGGCCTGCAAATTATTA
hNANOG	fw	TCCGGCACAGGAAGTTAGTT
	rev	AGACTCACCACCAGCAGCTT
hPRRX1	fw	AGACTGACCACTGGATGCTTTTG
	rev	GAAAAGCACATGGGAACCTGTC
hPDGFRa	fw	GGTGCCGTATGCAAATGGTT
	rev	CTTTGTCATGCAGAGTACCTCGATT
hTWIST1	fw	CTGCACATAAGGTCCGCTCAT
	rev	GGGCGCTTTGCTCAATTA
hZEB2	fw	GCTGATTACCATGAGAAAGGCTGT
	rev	GAGATTGACAGTCGCGTGGAG
hCXCL12	fw	TCCCCCAACATTCTTGCTCTT
	rev	GGCTTCAGTGTTTTCAGGATGG
hLGR5	fw	TGGCTGAGCTCGAATTCTTTTC
	rev	CCATCAGTAGGAATGCACTGTGAG
hmiR-21	fw	GTTATGCTGAAACTGGGCTGCT
	rev	CAAACCAAGAAATCCCCAGGTAG
